# Clinical situations for which 3D Printing is considered an appropriate representation or extension of data contained in a medical imaging examination: vascular conditions

**DOI:** 10.1186/s41205-023-00196-6

**Published:** 2023-11-30

**Authors:** Joonhyuk Lee, Seetharam C. Chadalavada, Anish Ghodadra, Arafat Ali, Elsa M. Arribas, Leonid Chepelev, Ciprian N. Ionita, Prashanth Ravi, Justin R. Ryan, Lumarie Santiago, Nicole Wake, Adnan M. Sheikh, Frank J. Rybicki, David H. Ballard

**Affiliations:** 1 Department of Radiology, University of Cincinnati Medical Center, Cincinnati, OH USA; 2 Department of Radiology, University of Pittsburgh Medical Center, Pittsburgh, PA USA; 3 Department of Bioengineering, University of Pittsburgh Medical Center, Pittsburgh, PA USA; 4 Department of Radiology, Henry Ford Health, Detroit, MI USA; 5 Department of Breast Imaging, The University of Texas MD Anderson Cancer Center, Houston, TX USA; 6 Joint Department of Medical Imaging, University of Toronto, Toronto, ON Canada; 7 Department of Biomedical Engineering, University at Buffalo, Buffalo, NY USA; 8 Webster Foundation 3D Innovations Lab, Rady Children’s Hospital, San Diego, CA USA; 9 Department of Neurological Surgery, University of California San Diego Health, San Diego, CA USA; 10 Department of Research and Scientific Affairs, GE HealthCare, New York, NY USA; 11 Center for Advanced Imaging Innovation and Research, Department of Radiology, NYU Langone Health, New York, NY USA; 12 Department of Radiology, University of British Columbia, Vancouver, Canada; 13 Department of Radiology, University of Arizona - Phoenix, Phoenix, AZ USA; 14 Mallinckrodt Institute of Radiology, Washington University School of Medicine, Saint Louis, MO USA

**Keywords:** 3D printing, Appropriateness, Quality, Radiology, Additive manufacturing, Rapid prototyping, Anatomic model, Vascular surgery, Vascular disease

## Abstract

**Background:**

Medical three-dimensional (3D) printing has demonstrated utility and value in anatomic models for vascular conditions. A writing group composed of the Radiological Society of North America (RSNA) Special Interest Group on 3D Printing (3DPSIG) provides appropriateness recommendations for vascular 3D printing indications.

**Methods:**

A structured literature search was conducted to identify all relevant articles using 3D printing technology associated with vascular indications. Each study was vetted by the authors and strength of evidence was assessed according to published appropriateness ratings.

**Results:**

Evidence-based recommendations for when 3D printing is appropriate are provided for the following areas: aneurysm, dissection, extremity vascular disease, other arterial diseases, acute venous thromboembolic disease, venous disorders, lymphedema, congenital vascular malformations, vascular trauma, vascular tumors, visceral vasculature for surgical planning, dialysis access, vascular research/development and modeling, and other vasculopathy. Recommendations are provided in accordance with strength of evidence of publications corresponding to each vascular condition combined with expert opinion from members of the 3DPSIG.

**Conclusion:**

This consensus appropriateness ratings document, created by the members of the 3DPSIG, provides an updated reference for clinical standards of 3D printing for the care of patients with vascular conditions.

**Supplementary Information:**

The online version contains supplementary material available at 10.1186/s41205-023-00196-6.

## Background

In 2018, the Radiological Society of North America (RSNA) Special Interest Group on Three-Dimensional Printing (3DPSIG) published appropriateness ratings for medical 3D printing and appropriateness for certain clinical scenarios including congenital heart disease, craniomaxillofacial pathologies, genitourinary pathologies, musculoskeletal pathologies, vascular pathologies, and breast pathologies [1]. Since then, there has been an expansion in the use of 3D printing to plan for vascular intervention, as well as more clinical reports. The purpose of this document is to update the clinical indications for 3D printing of vascular pathologies, and then vet, vote, and publish recommendations on their appropriateness.

## Methods

The 3DPSIG identified clinical situations for 3D printing of vascular conditions, and then provided recommendations for when 3D printing is considered usually appropriate, maybe appropriate, and rarely appropriate [2]. Strength of evidence was determined by literature review. The 3DPSIG Guidelines Chairperson managed the ratings of this document via a vote among 3DPSIG members. The results of the ratings follow the established 1–9 format (with 9 being the most appropriate):

- 1–3, red, rarely appropriate: There is a lack of a clear benefit or experience that shows an advantage over usual practice.
- 4–6, yellow, may be appropriate: There may be times when there is an advantage, but the data is lacking, or the benefits have not been fully defined.
- 7–9, green, usually appropriate: Data and experience shows an advantage to 3D printing as a method to represent and/or extend the value of data contained in the medical imaging examination.

Clinical scenarios were organized using standard categories of patients with vascular conditions [3]. A major treatise in vascular interventions served as a guide for search terms (Appendix 1), to ensure an exhaustive search [4–122]. Afterwards, an English language PubMed literature search through January 2022 and an appropriateness ratings document using standard categories for assessment were created. The supporting evidence was obtained through structured PubMed searches. From each search result, the relevant articles written in English were curated by consensus between physicians with expertise in 3D printing and vascular pathologies. Publications were deemed ineligible if they solely focused on bioprinting, virtual or augmented reality, or were review articles without new patient data. Neurovascular pathologies were excluded. All final included literature [4–122] and recommendations of this section were vetted and approved by vote of 3DPSIG members virtually at the July 20, 2022 3DPSIG Appropriateness Committee Meeting. Afterwards, the ratings and associated literature were posted on the 3DPSIG’s members-only online forum and comments could be made by 3DPSIG members for a 2-week period. All included studies were graded with a strength of evidence assessment, using as a methodology the assignment used by the American College of Radiology [2]. The paper represents the findings and conclusions of the 3DPSIG and does not represent an endorsement by the Radiological Society of North America (RSNA).

## Results

Table 1 provides evidence-based appropriateness ratings, supplemented by expert opinion when there was a paucity of peer-review data, to define and support the use of 3D printing for patients undergoing vascular intervention. The citations included in forming the appropriateness recommendations and the strength of evidence assessment are presented in Appendices 1 and 2 respectively.

**Table 1:** Appropriateness ratings for vascular conditions

Clinical Condition	Rating	References
**Aneurysms/Dissection- Central**		
Thoracic Aortic Aneurysm	**9**	4–13
Abdominal Aortic Aneurysm	**9**	14–46
Visceral Aneurysm/Pseudoaneurysm	**7**	47–53
Aortic Dissection	**6**	54–61
Coarctation	**5**	62–64
Penetrating Aortic Ulcer	**5**	65
**Extremity Vascular Disease**		
Upper Extremity Vascular Disease	**2**	
Lower Extremity Vascular Disease	**3**	68–69
**Acute Venous Thromboembolic Disease**		
Venous Thromboembolic Disease, acute	**2**	
Pulmonary Embolism, acute	**2**	70
**Venous Disorders**		
Varicose Veins	**1**	
Chronic Venous Insufficiency	**1**	
Nutcracker Syndrome	**5**	71–73
**Lymphedema**		
Lymphedema	**2**	74
**Congenital Vascular Malformations**		
Congenital Vascular Malformations	**7**	75–82
**Vascular Trauma**		
Vascular Trauma	**2**	
**Vascular Tumor**		
Primary Vascular Tumor	**6**	83
**Miscellaneous**		
Dialysis Fistulas/Grafts	**2**	84
**Vascular R&D and Modeling**		
Pre-clinical graft design	**5**	85–87
Pre-clinical stent design	**5**	88–98
Vascular Simulation (hemodynamics and interventions)	**9**	99–112
Modeling, other	**5**	113–119
**Vasculopathy, other**		
Atherosclerosis, other	**6**	120–124
Vasculitis, other	**1**	

## Discussion

### Aneurysms/dissection - central

Acute aortic syndrome includes patients who symptomatically present with chest or back pain, malignant hypertension, or hemodynamic instability. The most common specific diagnoses are aortic dissection, intramural hematoma, and unstable penetrating atherosclerotic ulcers. There are three general management option: open repair, endovascular treatment, or medical management with close imaging follow up. The literature supports 3D printing for intervention. The largest body of literature focuses on the management of aneurysms [4–6, 11–15, 19, 20, 24, 31, 33, 35, 37, 39–42, 45, 48, 49, 51–53], especially involving the abdominal aorta. There is additional literature on aortic dissection [58, 60, 61] and penetrating atherosclerotic ulcer. [65].

Extremity Vascular Disease.

Peripheral artery disease is common and presents with claudication. Patients are worked up with noninvasive studies and then imaging, the latter of which requires 3D visualization [123, 124]. Vascular intervention includes either percutaneous therapy or bypass. However, 3D printing to date has not been significantly involved in management. There are two studies where lower extremity anatomic models were used for surgical training or post-surgical assessment of alternative access or intervention [66, 67].

### Acute venous thromboembolic disease

Deep venous thrombosis and pulmonary embolism are common conditions where the diagnosis is typically confirmed by imaging. Treatment is most often medical (via anticoagulation), while some patients require more invasive therapies such as directed anticoagulation or thrombectomy. Patient-specific 3D printed anatomic models do not currently have a central role in managing most patients with these conditions. Our literature search yielded one study that demonstrated feasibility. In it, a 3D printed pulmonary arterial system was used to study flow dynamics during pulmonary angiography protocols, but the anatomic model was not used in patient care [68].

### Venous disorders

Venous disorders is a general term that includes chronic venous insufficiency, phlebitis, or varicose veins that typically cause lower extremity swelling and discomfort for patients. These are often managed medically and through lifestyle modifications, but can also be treated with minimally invasive endovascular interventions, e.g. radiofrequency vein ablation. 3D printed anatomic models do not appear in the literature, apart from patient-specific 3D printed extravascular stents to treat Nutcracker Syndrome [69–71].

### Lymphedema

Lymphedema presents with swelling, and in severe cases, restricted range of motion. Treatment is often conservative symptom management. One study developed a 3D printed lymphedema phantom for ultrasound tests, but it was not used in patient care [72].

### Congenital vascular malformations

Congenital vascular malformations have great variability in their presentation. The literature supports patient-specific 3D printed anatomic models for pre-intervention planning for Kommel’s diverticulum [73], double aortic arch [75, 78], and type II Abernathy malformation [77].

### Vascular trauma

Injury to local vascular structures can occur due to blunt or penetrating forces. Endovascular interventions can optimize treatment of such injury through minimally invasive approaches. At this time, no studies have been performed suggesting the utility of 3D printing in such cases.

### Vascular tumor

Resection of vascular tumors can be complicated when in anatomically sensitive locations or with extensive tissue involvement of the tumor. There is a single case series that used anatomic 3D printed models to retrospectively determine surgical margins of pulmonary artery sarcoma resections [81].

### Miscellaneous

Kidney failure leads to dialysis. Access via an arteriovenous fistula requires either open surgery or an endovascular approach. While there is no literature for pre-procedure patient-specific 3D printed anatomic models, one study used 3D printing for arteriovenous fistula surveillance post-operatively [82].

### Vascular R&D and modeling

Research and development in vascular surgery is continuously growing, especially in 3D printing applications. There are numerous pre-clinical studies detailing the involvement of 3D printing of stents and grafts, as well as modeling of vascular structure or flow dynamics [83–117].

### Vasculopathy, other

Atherosclerosis can impact vascular flow dynamics due to stenosis and impact patient outcomes. Several studies have used 3D printing to simulate these structural changes in a pre-clinical setting [118–122].

Inflammation of the vascular structures, or vasculitis, is a broad disease category, and 3D printed anatomic models have not been published in the management of vasculitis patients.

## Conclusion

This document updates clinical appropriateness for 3D printing for patients with vascular conditions. Adoption of common clinical standards regarding appropriate use, information and material management, and quality control are needed to ensure the greatest possible clinical benefit from 3D printing. With accruing evidence for utility and value in 3D printing, it is anticipated that this consensus appropriateness ratings document, created by the members of the 3DPSIG, will provide information that can be used for future clinical standards of 3D printing.

### Electronic supplementary material

Below is the link to the electronic supplementary material.

Supplementary Material 1

Supplementary Material 2

## Data Availability

The datasets used and/or analyzed during the current study are available from the corresponding author on reasonable request.
