# Supplementary material for: Clinical situations for which 3D Printing is considered an appropriate representation or extension of data contained in a medical imaging examination: vascular conditions
Source: 3D Print Med. 2023 Nov 30;9:34. doi: 10.1186/s41205-023-00196-6 (PMC10688120; doi:10.1186/s41205-023-00196-6)
Supplement: Supplementary file 1 — Supplementary Material 1 [file 41205_2023_196_MOESM1_ESM.docx]

**Appendix 1.** Search terms and categories to explore appropriate 3D printing applications for vascular conditions. This outline was guided by the chapters of a reputable text summarizing interventions in vascular conditions^1^.

1. Sidawy, A., Perler, B. *Rutherford's Vascular Surgery and Endovascular Therapy, 9th Edition*. 2018: Elsevier. 2832.

1. **Basic Science 1**
   1. Embryology and Developmental Anatomy
      1. ((3D Printing) AND (Vascular) AND (Embryology)) OR ((Rapid Prototyping) AND (Vascular) AND (Embryology))
   2. Atherosclerosis
      1. ((3D Printing) AND (Atherosclerosis)) OR ((Rapid Prototyping) AND (Atherosclerosis))
   3. Intimal Hyperplasia
      1. ((3D Printing) AND (Intimal Hyperplasia)) OR ((Rapid Prototyping) AND (Intimal Hyperplasia))
   4. Ischemia-Reperfusion
      1. ((3D Printing) AND (Ischemia)) OR ((Rapid Prototyping) AND (Ischemia)) OR ((3D Printing) AND (Reperfusion)) OR ((Rapid Prototyping) AND (Reperfusion))
   5. Arteriogenesis
      1. ((3D Printing) AND (Arteriogenesis)) OR ((Rapid Prototyping) AND (Arteriogenesis))
   6. Angiogenesis
      1. ((3D Printing) AND (Angiogenesis)) OR ((Rapid Prototyping) AND (Angiogenesis))
   7. Arterial Hemodynamics
      1. ((3D Printing) AND (Arterial) AND (Hemodynamics)) OR ((Rapid Prototyping) AND (Arterial) AND (Hemodynamics))
2. **Atherosclerotic Risk Factors**
   1. Atherosclerosis
      1. ((3D Printing) AND (Atherosclerosis)) OR ((Rapid Prototyping) AND (Atherosclerosis))
3. **Clinical and Vascular Laboratory Evaluation**
   1. Duplex Scan (Arterial or Venous)
      1. ((3D Printing) AND (Duplex Scan)) OR ((Rapid Prototyping) AND (Duplex Scan))
4. **Vascular Imaging**
   1. Radiation Safety
      1. ((3D Printing) AND (Radiation)) OR ((Rapid Prototyping) AND (Radiation))
   2. Arteriography
      1. ((3D Printing) AND (Arteriography)) OR ((Rapid Prototyping) AND (Arteriography))
   3. Venography
      1. ((3D Printing) AND (Venography)) OR ((Rapid Prototyping) AND (Venography))
   4. Computed Tomography
      1. ((3D Printing) AND ((Computed Tomography) OR (CT)) AND (vascular)) OR ((Rapid Prototyping) AND ((Computed Tomography) OR (CT)) AND (vascular))
   5. MRI
      1. ((3D Printing) AND ((MRI) OR (Magnetic Resonance Imaging)) AND (vascular)) OR ((Rapid Prototyping) AND ((MRI) OR (Magnetic Resonance Imaging)) AND (vascular))
   6. Vascular PET/CT
      1. ((3D Printing) AND ((PET) OR (Position Emission Tomography)) AND (vascular)) OR ((Rapid Prototyping) AND ((PET) OR (Position Emission Tomography)) AND (vascular))
   7. SPECT/CT
      1. ((3D Printing) AND ((SPECT) OR (Single-photon Emission Computed Tomography)) AND (vascular)) OR ((Rapid Prototyping) AND ((SPECT) OR (Single-photon Emission Computed Tomography)) AND (vascular))
   8. Intravascular US
      1. ((3D Printing) AND ((US) OR (Ultrasound)) AND ((vascular) OR (intravascular))) OR ((Rapid Prototyping) AND ((US) OR (Ultrasound)) AND ((vascular) OR (intravascular)))
5. **Perioperative Care**
   - 1. ((3D Printing) AND (perioperative) AND (vascular)) OR ((Rapid Prototyping) AND (perioperative) AND (vascular))
     2. ((3D Printing) AND (intraoperative) AND (vascular)) OR ((Rapid Prototyping) AND (intraoperative) AND (vascular))
6. **Bleeding and Clotting**
   1. Coagulopathy
      1. ((3D Printing) AND ((Coagulopathy) OR (Coagulation))) OR ((Rapid Prototyping) AND ((Coagulopathy) OR (Coagulation)))
   2. Hemorrhage
      1. ((3D Printing) AND (Hemorrhage)) OR ((Rapid Prototyping) AND (Hemorrhage))
   3. Hypercoaguable States
      1. ((3D Printing) AND (Hypercoaguable)) OR ((Rapid Prototyping) AND (Hypercoaguable))
7. **Complications**
   1. Graft Thrombosis/Infection
      1. ((3D Printing) AND (Graft) AND ((Thrombosis) OR (Infection))) OR ((Rapid Prototyping) AND (Graft) AND ((Thrombosis) OR (Infection)))
   2. Anastomotic Aneurysms
      1. ((3D Printing) AND (Anastomotic Aneurysm)) OR ((Rapid Prototyping) AND (Anastomotic Aneurysm))
   3. Aortoenteric Fistula
      1. ((3D Printing) AND (Aortoenteric Fistula)) OR ((Rapid Prototyping) AND (Aortoenteric Fistula))
8. **Technique**
   1. Thoracic and Thoracoabdominal Vascular Exposure
      1. ((3D Printing) AND ((Thoracic) OR (Thoracoabdominal)) AND (Vascular Exposure)) OR ((Rapid Prototyping) AND ((Thoracic) OR (Thoracoabdominal)) AND (Vascular Exposure))
   2. Abdominal Vascular Exposures
      1. ((3D Printing) AND (Abdominal) AND (Vascular Exposure)) OR ((Rapid Prototyping) AND (Abdominal) AND (Vascular Exposure))
   3. Cerebrovascular Exposure
      1. ((3D Printing) AND (Cerebrovascular Exposure)) OR ((Rapid Prototyping) AND (Cerebrovascular Exposure))
   4. Lower Extremity Arterial Exposure
      1. ((3D Printing) AND (Arterial Exposure) AND ((Lower Extremity) OR (leg))) OR ((Rapid Prototyping) AND ((Lower Extremity) OR (leg)))
   5. Upper Extremity Vascular Exposure
      1. ((3D Printing) AND (Vascular Exposure) AND ((Upper Extremity) OR (arm))) OR ((Rapid Prototyping) AND (Vascular Exposure) AND ((Upper Extremity) OR (arm)))
   6. Spinal Operative Exposure
      1. ((3D Printing) AND (Spinal Exposure)) OR ((Rapid Prototyping) AND (Spinal Exposure))
   7. Laparoscopic and Robotic Aortic Surgery
      1. ((3D Printing) AND (Laparoscopic Surgery)) OR ((Rapid Prototyping) AND (Laparoscopic Surgery))
      2. ((3D Printing) AND (Robotic Aortic Surgery)) OR ((Rapid Prototyping) AND (Robotic Aortic Surgery))
9. **Grafts and Devices**
   1. Autogenous/prosthetic/stent grafts
      1. ((3D Printing) AND (Graft) AND ((Autogenous) OR (Prosthetic) OR (Stent))) OR ((Rapid Prototyping) AND (Graft) AND ((Autogenous) OR (Prosthetic) OR (Stent)))
   2. Nonaortic Stents
      1. ((3D Printing) AND (Stents) NOT (Aortic)) OR ((Rapid Prototyping) AND (Graft) AND (Stents) NOT (Aortic))
10. **Abdominal Aortic and Iliac Aneurysms**
    1. Aortic, iliac, abdominal aneurysms
       1. ((3D Printing) AND (Aneurysm) OR ((Rapid Prototyping) AND (Aneurysm)) OR ((3D Printing) AND (Aneurysm) AND ((aortic) OR (iliac) OR (abdominal))) OR ((Rapid Prototyping) AND (Aneurysm) AND ((aortic) OR (iliac) OR (abdominal)))
11. **Thoracic and Thoracoabdominal Aortic Aneurysms and Dissections**
    1. Thoracic Endovascular Aortic Repair
       1. ((3D Printing) AND (Aortic Repair) AND (Thoracic)) OR ((Rapid Prototyping) AND (Aortic Repair) AND (Thoracic))
    2. Fenestrated and Branched Endograft Treatment
       1. ((3D Printing) AND (Endograft) AND ((Fenestrated) OR (Branched))) OR ((Rapid Prototyping) AND (Endograft) AND ((Fenestrated) OR (Branched)))
    3. Aortic Dissection
       1. ((3D Printing) AND (Aortic Dissection)) OR ((Rapid Prototyping) AND (Aortic Dissection))
    4. Penetrating Aortic Ulcer
       1. ((3D Printing) AND (Penetrating Aortic Ulcer)) OR ((Rapid Prototyping) AND (Penetrating Aortic Ulcer))
    5. Juxtarenal, paravisceral, thoracic, thoracoabdominal, aortic arch aneurysm
       1. ((3D Printing) AND (Aneurysm) OR ((Rapid Prototyping) AND (Aneurysm)) OR ((3D Printing) AND (Aneurysm) AND ((aortic) OR (thoracic) OR (juxtarenal) OR (paravisceral))) OR ((Rapid Prototyping) AND (Aneurysm) AND ((aortic) OR (thoracic) OR (juxtarenal) OR (paravisceral)))
12. **Peripheral and Splanchnic Aneurysms**
    1. Splanchnic Artery Aneurysm
       1. ((3D Printing) AND (Aneurysm) OR ((Rapid Prototyping) AND (Aneurysm)) OR ((3D Printing) AND (Aneurysm) AND (splanchnic)) AND (Aneurysm) AND (splanchnic))
13. **Cerebrovascular Diseases**
    1. Carotid Plaque
       1. ((3D Printing) AND (Carotid Plaque)) OR ((Rapid Prototyping) AND (Carotid Plaque))
    2. Nonatherosclerotic Carotid Artery Disease
       1. ((3D Printing) AND (Carotid Artery Disease) AND (nonatherosclerotic)) OR ((Rapid Prototyping) AND (Carotid Artery Disease) AND (nonatherosclerotic))
    3. Carotid Endarterectomy
       1. ((3D Printing) AND (Carotid Endoarterectomy)) OR ((Rapid Prototyping) AND (Carotid Endoarterectomy))
    4. Carotid Artery Stenting
       1. ((3D Printing) AND (Carotid Artery) AND (Stent)) OR ((Rapid Prototyping) AND (Carotid Artery) AND (Stent))
    5. Carotid Artery Dissection
       1. ((3D Printing) AND (Carotid Artery Dissection)) OR ((Rapid Prototyping) AND (Carotid Artery Dissection))
    6. Carotid Artery Aneurysm
       1. ((3D Printing) AND (Carotid Artery Aneurysm)) OR ((Rapid Prototyping) AND (Carotid Artery Aneurysm))
    7. Carotid Body Tumor
       1. ((3D Printing) AND (Carotid Body Tumor)) OR ((Rapid Prototyping) AND (Carotid Body Tumor))
    8. Cerebral Artery Dissection
       1. ((3D Printing) AND (Cerebral Artery Dissection)) OR ((Rapid Prototyping) AND (Cerebral Artery Dissection))
    9. Brachiocephalic Artery Disease
       1. ((3D Printing) AND (Brachiocephalic Artery Disease)) OR ((Rapid Prototyping) AND (Brachiocephalic Artery Disease))
14. **Acute Limb Ischemia**
    1. Acute Limb Ischemia
       1. ((3D Printing) AND (Acute Limb Ischemia)) OR ((Rapid Prototyping) AND (Acute Limb Ischemia))
    2. Compartment Syndrome
       1. ((3D Printing) AND (Compartment Syndrome)) OR ((Rapid Prototyping) AND (Compartment Syndrome))
    3. Atheromatous Embolization
       1. ((3D Printing) AND (Atheromatous Embolization)) OR ((Rapid Prototyping) AND (Atheromatous Embolization))
15. **Lower Extremity Chronic Arterial Disease**
    1. Lower Extremity Arterial Occlusive Disease
       1. ((3D Printing) AND (Arterial Occlusive Disease) AND ((Lower Extremity) OR (Legs))) OR ((Rapid Prototyping) AND (Arterial Occlusive Disease) AND ((Lower Extremity) OR (Legs)))
    2. Aortoiliac Disease
       1. Reconstruction, open extraanatomic Bypass
       2. ((3D Printing) AND (Aortoiliac Occlusive Disease)) OR ((Rapid Prototyping) AND (Aortoiliac Occlusive Disease))
       3. ((3D Printing) AND (Aortoiliac Occlusive Disease) AND (reconstruction)) OR ((Rapid Prototyping) AND (Aortoiliac Occlusive Disease) AND (reconstruction))
       4. ((3D Printing) AND (Extra-anatomic bypass)) OR ((Rapid Prototyping) AND (Extra-anatomic bypass))
    3. Infrainguinal Disease
       1. ((3D Printing) AND (Infrainguinal Occlusive Disease)) OR ((Rapid Prototyping) AND (Infrainguinal Occlusive Disease))
    4. Lower Extremity Amputations
       1. ((3D Printing) AND (Amputations) AND ((Lower Extremity) OR (legs))) OR ((Rapid Prototyping) AND (Amputations) AND ((Lower Extremity) OR (legs)))
16. **Diabetic Foot and its Management**
    1. Diabetic Foot
       1. ((3D Printing) AND (Diabetic Foot)) OR ((Rapid Prototyping) AND (Diabetic Foot))
17. **Upper Extremity Arterial Disease**
    1. Upper Extremity Arterial Disease
       1. ((3D Printing) AND (Upper Extremity Arterial Disease)) OR ((Rapid Prototyping) AND (Upper Extremity Arterial Disease)) OR ((3D Printing) AND (Vascular Disease) AND ((Upper Extremity) OR (arm))) OR ((Rapid Prototyping) AND (Vascular Disease) AND ((Upper Extremity) OR (arm)))
18. **Thoracic Outlet Syndrome**
    1. Thoracic Outlet Syndrome
       1. ((3D Printing) AND (Thoracic Outlet Syndrome)) OR ((Rapid Prototyping) AND (Thoracic Outlet Syndrome))
19. **Renovascular Disease**
    - 1. ((3D Printing) AND (Renovascular Disease)) OR ((Rapid Prototyping) AND (Renovascular disease)) OR ((3D Printing) AND (Renal Artery Stenosis)) OR ((Rapid Prototyping) AND (Renal Artery Stenosis))
20. **Mesenteric Vascular Disease**
    1. Mesenteric Arterial Dissection
       1. ((3D Printing) AND (Mesenteric Arterial Dissection)) OR ((Rapid Prototyping) AND (Mesenteric Arterial Dissection))
    2. Medical Arcuate Ligament Syndrome
       1. ((3D Printing) AND (Medical Arcuate Ligament Syndrome)) OR ((Rapid Prototyping) AND (Medical Arcuate Ligament Syndrome))
    3. Venous Thrombosis
       1. ((3D Printing) AND (Venous Thrombosis)) OR ((Rapid Prototyping) AND (Venous Thrombosis))
21. **Nonatherosclerotic Arterial Diseases**
    1. Vasculitis
       1. ((3D Printing) AND (Vasculitis)) OR ((Rapid Prototyping) AND (Vasculitis))
    2. Thromboangitis Obliterans (Buerger Disease)
       1. ((3D Printing) AND (Thromboangitis Obliterans)) OR ((Rapid Prototyping) AND (Thromboangitis Obliterans)) OR ((3D Printing) AND (Buerger Disease)) OR ((Rapid Prototyping) AND (Buerger Disease))
    3. Takayasu Disease
       1. ((3D Printing) AND (Takayasu Disease)) OR ((Rapid Prototyping) AND (Takayasu Disease))
    4. Raynaud Phenomenon
       1. ((3D Printing) AND (Raynaud Phenomenon)) OR ((Rapid Prototyping) AND (Raynaud Phenomenon))
    5. Fibromuscular Dysplasia
       1. ((3D Printing) AND (Fibromuscular Dysplasia)) OR ((Rapid Prototyping) AND (Fibromuscular Dysplasia))
    6. Nonatheromatous Popliteal Artery
       1. ((3D Printing) AND (Nonatheromatous Popliteal Artery)) OR ((Rapid Prototyping) AND (Nonatheromatous Popliteal Artery))
    7. Infected Arterial Aneurysms
       1. ((3D Printing) AND (Arterial Aneurysms) AND (infection)) OR ((Rapid Prototyping) AND (Arterial Aneurysms) AND (infection))
22. **Acute Venous Thromboembolic Disease**
    1. Acute Deep Venous Thrombosis
       1. ((3D Printing) AND ((Deep Vein Thrombosis) OR (DVT))) OR ((Rapid Prototyping) ((Deep Vein Thrombosis) OR (DVT)))
    2. Venous Thromboembolic Disease
       1. ((3D Printing) AND (Venous Thromboembolic Disease)) OR ((Rapid Prototyping) AND (Venous Thromboembolic Disease))
    3. Catheter-related Venous Thrombosis
       1. ((3D Printing) AND (Venous Thrombosis) AND (catheter)) OR ((Rapid Prototyping) AND (Venous Thrombosis) AND (catheter))
    4. Superficial Thrombophlebitis
       1. ((3D Printing) AND (Superficial Thrombophlebitis)) OR ((Rapid Prototyping) AND (Superficial Thrombophlebitis))
    5. Pulmonary Embolism
       1. ((3D Printing) AND ((Pulmonary Embolism) OR (PE))) OR ((Rapid Prototyping) AND ((Pulmonary Embolism) OR (PE)))
    6. Vena Cava Interruption
       1. ((3D Printing) AND (Vena Cava Interruption)) OR ((Rapid Prototyping) AND (Vena Cava Interruption))
    7. Compartment Syndrome and Venous Gangrene
       1. ((3D Printing) AND (Venous Gangrene)) OR ((Rapid Prototyping) AND (Venous Gangrene))
23. **Chronic Venous Disorders**
    1. Varicose Veins
       1. ((3D Printing) AND (Varicose Veins)) OR ((Rapid Prototyping) AND (Varicose Veins))
    2. Chronic Venous Insufficiency
       1. Perforator Vein Incompetence
       2. Deep Vein Valve Reconstruction
       3. ((3D Printing) AND (Chronic Venous Insufficiency)) OR ((Rapid Prototyping) AND (Chronic Venous Insufficiency))
       4. ((3D Printing) AND (Deep Vein Valve) AND ((Repair) OR (Reconstruction))) OR ((Rapid Prototyping) AND (Deep Vein Valve) AND ((Repair) OR (Reconstruction)))
       5. ((3D Printing) AND (Perforator Vein) AND ((Incompetence) OR (Insufficiency))) OR ((Rapid Prototyping) AND (Perforator Vein) AND ((Incompetence) OR (Insufficiency)))
    3. Iliocaval Venous Obstruction
       1. ((3D Printing) AND (Iliocaval Venous Obstruction)) OR ((Rapid Prototyping) AND (Iliocaval Venous Obstruction))
24. **Miscellaneous Venous Conditions**
    1. Superior Vena Cava Occlusion
       1. ((3D Printing) AND ((Superior Vena Cava) OR (SVC)) AND ((Occlusion) OR (Obstruction))) OR ((Rapid Prototyping) AND ((Superior Vena Cava) OR (SVC)) AND ((Occlusion) OR (Obstruction)))
    2. Absence of Inferior Vena Cava
       1. ((3D Printing) AND ((Inferior Vena Cava) OR (IVC)) AND (absence)) OR ((Rapid Prototyping) AND ((Inferior Vena Cava) OR (IVC)) AND (absence))
    3. Portal Hypertension
       1. ((3D Printing) AND (Portal Hypertension)) OR ((Rapid Prototyping) AND (Portal Hypertension))
    4. Nutcracker Syndrome
       1. ((3D Printing) AND (Nutcracker Syndrome)) OR ((Rapid Prototyping) AND (Nutcracker Syndrome))
25. **Lymphedema**
    - 1. ((3D Printing) AND (Lymphedema)) OR ((Rapid Prototyping) AND (Lymphedema))
26. **Vascular Malformations**
    1. Congenital Vascular Malformations
       1. ((3D Printing) AND (Vascular Malformation) AND (congenital)) OR ((Rapid Prototyping) AND (Vascular Malformation) AND (congenital))
    2. Acquired Arteriovenous Fistulas
       1. ((3D Printing) AND ((Acquired Arteriovenous Fistula) OR (AV fistula))) OR ((Rapid Prototyping) AND ((Acquired Arteriovenous Fistula) OR (AV fistula)))
27. **Hemodialysis Access**
    1. Hemodialysis access
       1. ((3D Printing) AND (Hemodialysis)) OR ((Rapid Prototyping) AND (Hemodialysis))
28. **Vascular Trauma**
    1. Vascular Trauma
       1. ((3D Printing) AND (Vascular Trauma)) OR ((Rapid Prototyping) AND (Vascular Trauma)) OR ((3D Printing) AND (Extremity Vascular Trauma)) OR ((Rapid Prototyping) AND (Extremity Vascular Trauma))
29. **Special Issues in Pediatric Vascular Surgery**
    1. Pediatric Vascular Tumors
       1. ((3D Printing) AND ((pediatric) OR (infants) OR (adolescents)) AND (vascular) AND ((tumor) OR (neoplasia))) OR ((Rapid Prototyping) AND ((pediatric) OR (infants) OR (adolescents)) AND (vascular) AND ((tumor) OR (neoplasia)))
30. **Miscellaneous Conditions**
    1. Erectile Dysfunction
       1. ((3D Printing) AND (Erectile Dysfunction)) OR ((Rapid Prototyping) AND (Erectile Dysfunction))
    2. Complex Regional Pain Syndrome
       1. ((3D Printing) AND (Complex Regional Pain Syndrome)) OR ((Rapid Prototyping) AND (Complex Regional Pain Syndrome))
    3. Vascular Tumors
       1. ((3D Printing) AND (vascular) AND ((tumor) OR (neoplasia))) OR ((Rapid Prototyping) AND (vascular) AND ((tumor) OR (neoplasia)))
31. **Business of Vascular Surgery**
    1. N/A
